# Development of Psychosocial Distress in Cancer Survivors and Its Potential Prognostic Impact on Survival: A Scoping Review

**DOI:** 10.1002/pon.70488

**Published:** 2026-05-15

**Authors:** Yifeng Gao, Chien‐Tzu Lee, Melissa S. Y. Thong, Daniela Doege, Volker Arndt

**Affiliations:** ^1^ Cancer Survivorship Outcomes and Epidemiology German Cancer Research Center (DKFZ) Heidelberg Germany; ^2^ Medical Faculty Heidelberg University Heidelberg Germany

**Keywords:** anxiety, cancer survivors, depression, longitudinal studies, psychological distress, survival, survivorship

## Abstract

**Background:**

While single‐point assessments of psychosocial distress have been associated with survival outcomes, investigations examining longitudinal changes in psychosocial distress and survival outcomes remain limited.

**Aims:**

This scoping review evaluates existing literature on longitudinal distress patterns and their association with survival outcomes.

**Methods:**

A comprehensive search was performed in PubMed, Web of Science, and PsycInfo databases up to January 2025. Studies were eligible if they included adult cancer survivors, measured psychological distress using validated instruments at minimum two time points, and employed longitudinal cohort designs. Quality assessment used a modified Newcastle‐Ottawa Scale. A narrative synthesis was performed due to study heterogeneity.

**Results:**

Of 5028 records screened, 41 studies met criteria, representing diverse geographical regions and cancer types (sample sizes: 74–6054 participants). Eleven examined distress‐survival associations; 30 focused on longitudinal changes. Studies identified 2–6 distinct trajectory patterns, with most survivors maintaining low distress levels while a minority experienced chronically elevated symptoms. Persistent or worsening psychological distress was consistently associated with worse survival outcomes across cancer types, while survivors recovering from initial symptoms showed survival rates comparable to those never experiencing distress.

**Conclusions:**

Longitudinal psychological distress patterns may carry prognostic value in cancer survivors, with persistent or worsening symptoms generally associated with worse survival outcomes, though effect sizes varied and findings were not uniformly consistent. These findings suggest a potential role for longitudinal distress monitoring during survivorship care. Whether modifying distress trajectories through psychosocial interventions can improve survival remains unclear and warrants further investigation.

## 1 Introduction

Cancer survivorship has become a major public health priority as advances in early detection, diagnostic precision, and treatment modalities have significantly improved the prognosis and long‐term survival of people diagnosed with malignancies [1]. There were an estimated 18.1 million US cancer survivors as of January 1, 2022, with 70% having lived 5 years or more after diagnosis and 11% having lived 25 years or more after diagnosis [2]. In Europe, 14.8 million people were estimated to be alive more than 5 years after diagnosis and 9.1 million people more than 10 years after diagnosis in 2020, representing an increasing proportion of the cancer survivor population [3].

Psychological impact of cancer extends well beyond the acute diagnosis and treatment phases [4]. Psychological distress—manifesting as depression, anxiety, fear of cancer recurrence (FCR), or emotional dysfunction in general—remains a significant concern for many survivors, even years after treatment completion [5, 6, 7]. When compared with the general population, the prevalence of mental disorders and emotional distress among cancer survivors substantially exceeds that of the general population even in the long‐term [8, 9].

This persistent distress is not merely a co‐occurring issue but may act as an independent prognostic factor. Longitudinal changes in psychological distress patterns may influence the disease trajectory for cancer survivors itself or through multiple pathways, including compromised immune responses and inflammation processes, ultimately affecting disease progression, recurrence and survival. While extensive research has examined one‐time assessments of psychological distress in relation to survival outcomes, with several systematic reviews and meta‐analyses identifying psychological distress as independently associated with poor overall and progression‐free survival in cancer survivors [10, 11], and substantial research has characterized distinct trajectories or change patterns of psychosocial adaptation over time [12, 13], few studies have combined these approaches. Investigations examining the relationship between the dynamic course of psychosocial distress and survival outcomes remain limited. The chronic nature of distress and its cumulative effects over time underscore the need for longitudinal investigations that move beyond one‐time assessments.

Despite growing interest in this area, there is a scarcity of longitudinal studies with long periods (more than 5 or more) post‐diagnosis that comprehensively evaluate the change patterns of psychosocial factors on survival of cancer survivors. Furthermore, the heterogeneity in study populations, assessment instruments, analytical approaches, and outcome definitions complicates interpretation and integration of findings across studies. Therefore, this scoping review aims to map and evaluate the existing literature on longitudinal changes in psychosocial distress among cancer survivors and their potential association with survival outcomes. Specifically, we seek to: (1) identify common patterns of change in psychological distress over time among cancer survivors; (2) synthesize the evidence regarding associations between changes in psychological distress and survival outcomes; and (3) identify research gaps and methodological considerations to inform future studies in this field.

## 2 Methods

We used the Preferred Reporting Items for Systematic Reviews and Meta‐Analyses extension for Scoping Reviews (PRISMA‐ScR) checklist to report the review findings [14]. The protocol for this scoping review is publicly accessible online on the Open Science Framework: OSF|Development of Psychosocial Distress and Its Potential Prognostic Impact on Survival in Cancer Survivors: A Scoping Review.

### 2.1 Information Sources and Search Strategy

A comprehensive search was performed in three electronic databases: PubMed, Web of Science, and PsycInfo. The search strategy combined terms related to psychological/psychosocial distress (including depression, anxiety, FCR), cancer survivors/patients, mortality/survival outcomes, and longitudinal/cohort study designs. Both Medical Subject Headings (MeSH) terms and free‐text keywords were used. The full search strategies for each database are provided in the Supplementary materials.

### 2.2 Eligibility Criteria

Studies were eligible for inclusion if they met the following criteria: included adult cancer survivors (individuals diagnosed with any type of malignancy during adulthood); measured psychological distress using validated instruments at minimum two time points; reported on changes in psychological distress over time and/or the association between these changes and survival outcomes; employed a longitudinal cohort design; and were published in English to January 2025. Studies were excluded if they focused on childhood or adolescent cancer, employed a cross‐sectional design or were randomized controlled trials, did not report on longitudinal changes in psychological distress, or were not published as full‐text peer reviewed articles (including abstracts, dissertations, conference proceedings, reports, or other gray literature).

### 2.3 Data Extraction

Two authors (Y.G. and C.‐T.L.) independently reviewed the articles to determine whether they met the predetermined eligibility criteria. Any disagreements were resolved through discussion with involvement of a third author (D.D.).

From each included study, the following data were systematically extracted: first author, publication year, study design, country/region of study, data sources, cancer type, sample size, participant demographic characteristics, follow‐up duration, number of assessment points, instruments used to measure psychological distress, outcome variables, statistical methods employed, and main findings.

### 2.4 Risk of Bias Assessment

The methodological quality of the included studies was assessed using the Newcastle‐Ottawa Scale (NOS) for cohort studies. This validated tool was selected because it specifically addresses the quality domains relevant to longitudinal cohort studies. The standard NOS evaluates studies across three domains: selection of study groups, comparability of groups, and ascertainment of exposure or outcome, with a maximum total score of 9 stars. However, given that we included studies that focused on the longitudinal changes in psychological distress within cancer survivor cohorts, the inclusion of a non‐exposed cohort was not a methodological requirement aligned with the objectives of our scoping review. Therefore, a modified NOS with a maximum total score of 8 stars was applied, excluding the “selection of the non‐exposed cohort” criterion. When assessing the comparability between groups, stricter criteria were applied when determining the importance of covariates included for adjustment in the models. Age, sex, education level, income, and cancer stage of cancer survivors are all considered “most important,” while factors such as comorbidity or other psychological variables are considered “additionally important.” Studies were classified as high quality (6–8 stars), moderate quality (3–5 stars), or low quality (0–2 stars) [15]. Two reviewers (Gao and Lee) conducted the quality assessment independently.

### 2.5 Data Synthesis

Due to the heterogeneity in study populations, psychological distress measures, follow‐up periods, and analytical approaches, a narrative synthesis was conducted rather than a meta‐analysis. The synthesis was structured according to characteristics of the included studies, patterns of change in psychological distress over time among cancer survivors, and associations between changes in psychological distress and survival outcomes. The analysis was further stratified by follow‐up duration to explore potential sources of heterogeneity in psychological distress trajectories and their prognostic significance.

## 3 Results

### 3.1 Study Selection

**FIGURE 1:**
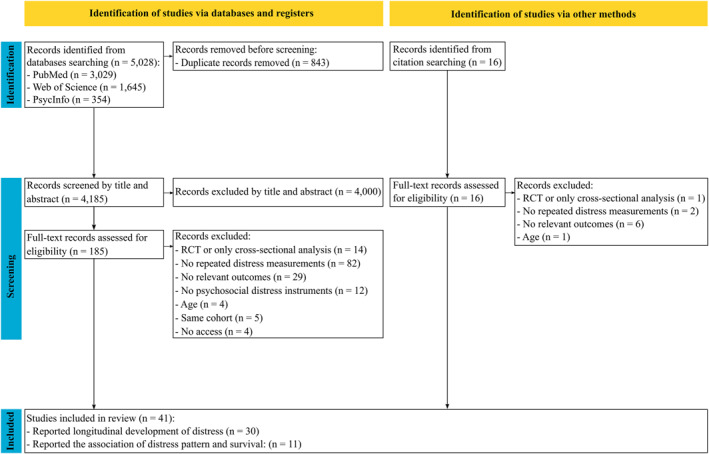
PRISMA Flow chart of the study selection process.

The systematic search of three databases yielded a total of 5028 records (PubMed: *n* = 3029; Web of Science: *n* = 1645; PsycInfo: *n* = 354). After removing 843 duplicates, 4185 records remained for title and abstract screening. This screening resulted in the exclusion of 4000 records that did not meet the inclusion criteria. The remaining 185 articles underwent full‐text review, which led to the exclusion of 149 articles for various reasons (see Figure 1). Additionally, 16 potentially eligible records were identified through citation searching. After full‐text review of these additional records, 10 were excluded. This process resulted in 41 studies meeting all eligibility criteria for inclusion in this scoping review. Of these, 11 examined both longitudinal changes in psychological distress and their association with survival outcomes, while 30 focused solely on longitudinal changes in psychological distress over time.

### 3.2 Risk of Bias Assessment Results

Overall, the majority of studies were of high quality, with 39 (95.1%) scoring between 6–8 stars, while 2 (4.9%) were of moderate quality. No studies were rated as low quality. Among the 11 studies examining both distress changes and its relationship with survival, 10 (90.9%) were of high quality, with 4 studies (36.4%) achieving the maximum score, and only 1 study (9.1%) were of moderate quality. Common strengths across studies included appropriate sample selection, validated measurement tools for assessing psychological distress, and adequate follow‐up duration. The limitations included limited representativeness or generalizability and insufficient control for additional important confounding variables. This quality assessment indicates that the body of evidence included in this scoping review is predominantly of high methodological quality, providing a solid foundation for the synthesis and interpretation of findings regarding psychological distress trajectories and their potential prognostic impact among cancer survivors (Table 1 and Supporting Information S1: Table S1).

**TABLE 1:** Quality assessment of 11 studies on the association between longitudinal psychological distress and survival in cancer survivors by modified Newcastle‐Ottawa quality assessment scale (NOS)^a^ for cohort studies.

Study	Selection (3 stars)			Comparability (2 stars)	Outcome (3 stars)			Modified final score (8 stars)
	Representativeness of the exposed cohort	Ascertainment of exposure	Outcome of interest was not present at start of study	Comparability of cohorts on the basis of the design or analysis^b^	Assessment of outcome	Was follow‐up long enough for outcomes to occur	Adequacy of follow up of cohorts	
Borza et al. 2022 [16]	*	*	*	**	*		*	7
K.W. Brown et al. 2003 [17]		*	*	**	*	*	*	7
Hasegawa et al., 2019 [18]		*	*	*	*	*	*	6
Jansen et al. 2018 [19]	*	*	*	*	*	*	*	7
Jarnagin et al. [20]		*	*	*	*		*	5
Lei et al. 2023 [21]	*	*	*	**	*	*	*	8
Orive et al. 2023 [22]	*	*	*	**	*	*	*	8
Pandit et al. 2022 [23]	*	*	*	*	*	*	*	7
Sanghvi et al. 2024 [24]	*	*	*	**	*	*	*	8
Sullivan et al. 2016 [25]	*	*	*	**	*	*	*	8
Zhou and Sun 2021 [26]		*	*	*	*	*	*	6

^a^ The standard NOS (max 9 stars) assesses selection, comparability, and exposure/outcome. For studies on longitudinal distress in cancer survivor cohorts, a non‐exposed group was not required; therefore, a modified NOS (max 8 stars) was applied, excluding this criterion.

^b^ This section consists of 2 questions accounting for 1 star per question: “Study controls for most important factor” and “Study controls for additional important factor”.

### 3.3 Summarized Characteristics of Included Studies

The 41 studies included in this review represented diverse geographical regions. All studies employed a longitudinal cohort design, with participants recruited through various sources. Most studies (*n* = 33) examined specific solid tumors, one study investigated hematological malignancy, and seven investigated mixed cancers. The range of sample sizes (74–6054 participants) and age (mean age approximately 40–70 years) varied considerably across studies, reflecting the heterogeneity of the study targets. The duration of psychosocial distress follow‐up (time between first and last distress assessment) also varied substantially across studies, ranging from as short as 60 days to as long as 10 years. Among the 11 studies examining survival outcomes, the follow‐up time for mortality assessment (from initial distress measurement to death date/censor time) ranged from 13.5 months to 10 years, with a median follow‐up of approximately 5 years (Table 2).

**TABLE 2:** Summarized characteristics of all included studies.

	Study only focused on longitudinal change pattern (*n* = 30)	Study examining distress change pattern‐survival relationship (*n* = 11)
Cancer type		
Breast	12	1
Colorectal	4	2
Other gastrointestinal (esophageal, gastric, pancreaticobiliary)	2	1
Brain	2	NA
Lung	1	1
Melanoma	2	NA
Gynecological	2	NA
Head and neck	1	1
Bladder	NA	1
Hematological	NA	1
Mixed	4	3
Geographical region		
North America	10	6
Europe	11	3
East Asia	6	2
Australia	3	NA
Data source		
Cancer registry	3	2
Population‐based study cohort	6	3
Single‐center	9	4
Multi‐center	10	2
Chart review	1	NA
Convenience sample	1	NA
Sample size^a^	74–2625	134–6054
Mean age (years)^b^	45.4–69.4	55.8–76.9
Length of follow‐up (FU)^c^		
FU spanning assessments of psychosocial distress	60 days–10 years	1 month–6 years
FU spanning survival time	NA	13.5 months–10 years

^a^ Sample size determination varied across studies. Some studies included only participants with complete data at all assessment time points, while others included all participants who completed baseline assessments for analysis. Summary statistics are reported according to the text.

^b^ Mean age was not fully presented in few studies. Summary statistics are based only on studies that provided complete age data.

^c^ Information about length of follow‐up are not fully presented in few studies that focused on the acute treatment phase. Summary values reflect only studies that provided clear follow‐up time information.

### 3.4 Methodological Approaches of Included Studies

#### 3.4.1 Measures of Psychological Distress

Psychological distress was assessed using diverse validated instruments across the 41 included studies. Depression was the most frequently measured construct (*n* = 21), followed by overall distress (*n* = 13), anxiety (*n* = 9), FCR (*n* = 7), and mental well‐being (*n* = 2).

A range of validated instruments was employed to measure these constructs. For depression assessment, the Hospital Anxiety and Depression Scale‐Depression subscale (HADS‐D) was the most commonly used instrument, with a cut‐off > 7 for clinically elevated depression, appearing in 9 studies [9, 19, 26, 27, 28, 29, 30, 31, 32]. Other frequently used instruments included the Center for Epidemiologic Studies Depression Scale (CES‐D) [17, 24, 25, 33, 34, 35] and the Patient Health Questionnaire‐Depression subscale (PHQ‐D) [18, 20]. Only one study used International Classification of Diseases, 9th Revision, Clinical Modification (ICD‐9) diagnoses instead of self‐report [21]. For anxiety measurements, the HADS‐Anxiety subscale (HADS‐A) was most frequently employed [9, 17, 26, 27, 30, 31, 36], while two studies used the State Trait Anxiety Inventory (STAI) [32, 33]. FCR measurements showed considerable variation, with two studies using the Fear of Cancer Recurrence Inventory (FCRI) [37, 38] and two studies using the Cancer Worry Scale (CWS) [39, 40]. General distress was measured using various instruments including the Distress Thermometer (DT) [27, 41, 42, 43], Brief Symptom Inventory (BSI‐18) [44, 45], 12‐item General Health Questionnaire (GHQ) [46], and Edmonton Symptom Assessment System‐revised (ESAS‐r) [47]. Four studies defined distress as combined anxiety and depression scores using HADS [22, 48, 49, 50], and one study used PHQ‐4 to measure anxiety and depression as a composed distress variable [51]. Two studies used the SF‐36 Mental Component Score (MCS) for overall mental well‐being [23, 52], which employed reverse scoring where higher scores indicated better mental health.

Detailed table of the instruments and their according cutoff scores employed in the studies is available in Table 3 and Supporting Information S1: Table S2.

**TABLE 3:** Characteristics of 11 included studies on the association between longitudinal psychological distress and survival in cancer survivors.

Main author, year, Country	Design	Data source	Cancer type	Population^a^	Distress instrument (cut‐off values)	Sample size^b^	Assessments	Follow‐up (FU) length	Survival outcome	Modeling
Borza et al. 2022 [16] Norway	Prospective cohort	8 oncology outpatient clinics	Mixed	‐ Age: ≥ 70 years; mean age: 76.9 ± 5.1 years ‐ Female: 44%	Depression: GDS‐15 (continuous)	284	3: At inclusion; 4 months FU; 12 months FU	Up to 2 years	Overall survival	‐ For change patterns: Generalized linear mixed model and linear mixed model with the same fixed and random effects; ‐ For survival analysis: Unadjusted extended cox regression model
K.W. Brown et al. 2003 [17] Canada	Retrospective cohort	Jewish general hospital	Mixed	‐ Mean age: 55.8 ± 12.5 years for the alive; 57.1 ± 13.6 years for the deceased ‐ Female: 75%	Depression and overall distress: 20 items CES‐D (continuous)	205	4: After diagnosis; 4 months; 8 months; 12 months	Up to 10 years	Cancer‐specific survival	‐ For change patterns: Repeated measures ANOVA; ‐ For survival analysis: Multivariable cox proportional hazards models
Hasegawa et al. 2019 [18] Japan	Prospective cohort	Department of hematology and oncology, Nagoya city university hospital	Hematological (malignant lymphoma or multiple myeloma)	‐ Disease stages: Ann arbor stages I to IV for malignant lymphoma; ISS stages I to III for multiple myeloma	Depression: PHQ‐9 (cut‐off = 9/10)	255	2: Before chemotherapy; 1 month later	Median 1268 days for censored patients	Overall survival	‐ For change patterns: Categorized by “new onset,” “remission,” and “persistent;” ‐ For survival analysis: Multivariate cox proportional hazards model
Jansen et al. 2018 [19] UK	Retrospective cohort	The head and neck 5000 study	Head and neck	‐ Age: ≥ 18 years ‐ Tumor stages: I To IV	Depression: HADS‐D (cut‐off > 7)	1217	3: Pre‐treatment; 4 months; 12 months	Median 1046 days	Overall survival	‐ For change patterns: Categorized by “never depression symptoms,” “recovered from depression symptoms,” and “persistent/recurrent/late depression symptoms;” ‐ For survival analysis: Cox regression analyses
Jarnagin et al. 2023 [20] USA	Prospective cohort	Massachusetts general hospital cancer center	Pancreaticobiliary, colorectal, and gastroesophageal	‐ Median age: 64 (28–84) ‐ Female: 33.8% ‐ Disease stage: Advanced/metastatic	Depression and anxiety: PHQ‐4 (continuous)	134	2: At chemotherapy initiation; 1 month	Median 13.5 months	Overall survival; progression‐free survival	‐ For change patterns: Change values between two assessments; ‐ For survival analysis: Cox regression model
Lei et al. 2023 [21] USA	Retrospective cohort	The health administrative claims‐linked Kentucky cancer registry	Breast	‐ Median age: 70 (23–101) years ‐ Disease stages: I To IV	Depression: ICD‐9‐CM (dichotomous)	6054	2: 1 year before diagnosis; 1 year after diagnosis	Median 47.8 months	Overall survival	‐ For change patterns: Categorized by “no depression,” “pre‐diagnosis only,” “post‐diagnosis only” and “persistent depression;” ‐ For survival analysis: Multivariate cox regression models
Orive et al. 2023 [22] Spain	Prospective cohort	19 public hospitals representing 9 provinces	Colorectal	‐ Female: 36.67% ‐ pTNM stages: 0 to IV	Depression and anxiety: HADS‐A and HADS‐D (cut‐off > 7 for each)	2531	5: Pre‐surgery; 1 year; 2 years; 3 years; 5 years	Up to 5 years	Overall survival	Multivariate cox proportional models with HADS scores as time‐dependent predictor
Pandit et al. 2022 [23] USA	Retrospective cohort	SEER cancer registry for death linked with MHOS for PROs	Bladder	‐ Mean age: 76.3 ± 6.5 years ‐ Female: 26.5%	Mental well‐being: SF‐36/VR‐12 MCS (continuous)	438	2: Before diagnosis; after diagnosis	Median 89 months	Overall survival	‐ For change patterns: Categorized by “pre‐diagnosis only,” “post‐diagnosis only” and “pre‐to‐post MCS;” ‐ For survival analysis: Multivariate cox proportional hazards models
Sanghvi et al. 2024 [24] USA	Retrospective cohort	The health and retirement study	Mixed	‐ Mean age: 70.04 ± 9.89 ‐ Female: 46.41%	Depression: CES‐D (continuous)	2342	4: Before cancer; 2 years; 4 years; 6 years;	Up to 6 years	Overall survival	‐ For change patterns: Latent growth mixture modeling; ‐ For distress pattern‐survival analysis: Logistic regression to assess trajectories as predictors of mortality risk
Sullivan et al. 2016 [25] USA	Prospective cohort	The national cancer institute‐funded cancer care outcomes research and surveillance study	Lung	‐ Age: ≥ 21 years, 57% older than 65 years ‐ Female: 45% ‐ Disease stage: I To IV	Depression: CES‐D (cut‐off ≥ 4)	1790	2: 5 months after diagnosis; 12 months after diagnosis	Up to 8 years	Overall survival	‐ For change patterns: Categorized by “never,” “new‐onset,” “remission” and “persistent;” ‐ For survival analysis: Cox's proportional‐hazards regression models
Zhou and Sun 2021 [26] China	Prospective cohort	The 2nd affiliated hospital of harbin medical university	Colorectal	‐ Mean age: 63.7 ± 10.2 years ‐ Female: 43.7% ‐ TNM stage: I–III	Anxiety and depression: HADS‐A and HADS‐D (cut‐off ≥ 8 for each)	302	13: At discharge; every 3 months	Up to 36 months	Overall survival	‐ For change patterns: Repeated measures ANOVA; ‐ For survival analysis: Univariate and multivariate cox's proportional hazard regression

Abbreviations: CES‐D, Center for Epidemiologic Studies Depression Scale; GDS‐15, Geriatric Depression Scale—15 Items; HADS, Hospital Anxiety and Depression Scale; HADS‐A, Hospital Anxiety and Depression Scale—Anxiety Subscale; HADS‐D, Hospital Anxiety and Depression Scale—Depression Subscale; ICD‐9‐CM, International Classification of Diseases, 9th Revision, Clinical Modification; MCS, Mental Component Score; PHQ‐4, Patient Health Questionnaire—4 Items; PHQ‐9, Patient Health Questionnaire—9 Items; SF‐36, Short Form—36 Health Survey; VR‐12, Veterans RAND 12‐Item Health Survey.

^a^ Mean age was not fully presented in few studies. Summary statistics are based only on studies that provided complete age data.

^b^ The way of determining sample size used for analysis varied across studies. Some studies included only participants with complete data at all assessment time points, while others included all participants who completed baseline assessments for analysis. Summary statistics are reported according to the text.

#### 3.4.2 Assessment Timing and Frequency

The timing and frequency of distress assessments varied substantially across studies. The first assessment point ranged from pre‐diagnosis to several years post‐diagnosis, with the majority of studies initiating assessment within the first year after diagnosis. The number of assessment points ranged from 2 to 13, with most studies including between 2 and 4 assessment points. Studies with extended follow‐up periods (> 5 years) generally employed fewer assessment points with longer intervals between assessments, while studies with shorter follow‐up periods (< 2 years) typically used more frequent assessments.

#### 3.4.3 Statistical Methods

Various statistical approaches were employed to analyze longitudinal changes in psychological distress. The most common methods included growth mixture modeling (GMM) [28, 31, 33, 34, 35, 36, 40, 41, 42, 44, 45, 53], latent class growth analysis (LCGA) [39, 50, 54, 55], and group‐based trajectory modeling (GBTM) [27, 29, 43, 48, 51, 52]. Several studies also used simpler approaches such as repeated measures ANOVA between time points [32, 49, 53] or linear mixed‐effects models [9, 37, 38, 54] to evaluate the overall changes in distress symptoms over time.

In studies examining associations with survival outcomes, the employment of Cox proportional hazards models was universal, with varying approaches to incorporating the longitudinal distress data. Only one study used trajectory group membership as a predictor [24], while four studies incorporated time‐varying covariates to capture changes in distress over time [16, 17, 22, 26]. Six of 11 studies examining associations with survival outcomes used simpler approaches such as categorizing survivors based on whether their distress increased, decreased, or remained stable over time [18, 19, 20, 21, 23, 25].

### 3.5 Longitudinal Trajectories of Psychological Distress in Cancer Survivors

Across the 30 studies examining longitudinal development of psychological distress using latent trajectory modeling methods, 2–6 distinct trajectory patterns were identified in most studies. The studies that did not apply trajectory grouping revealed a common, gradually worsening trend in psychosocial distress (including depression, anxiety, FCR, and overall general distress) during the follow‐up period [31, 32, 37, 38]. In those studies that only looked at the change throughout chemotherapy, end of treatment was usually found to be a time point where the psychological distress improved significantly [32, 40]. When compared with the population norm or control group, some studies found that the distress level was comparable in survivors and the controls after 2 years post diagnosis [52], but one study found that the distress level was higher in cancer survivors [9].

The most frequently identified trajectory patterns across studies were characterized by consistently minimal psychological symptoms and consistently elevated symptoms throughout the cancer survivorship continuum. The “consistent low distress” or “resilient” pattern was documented in 21 out of 30 studies, of which 18 consistently identified this pattern as the largest group, regardless of cancer type, follow‐up duration, or number of trajectories. The “high stable” or “persistently elevated” distress trajectory was reported in 20 studies and it typically classified a smaller proportion of cancer survivors with most studies focusing on specific cancer types identifying a depression, anxiety, FCR or general distress rates under 20%. However, in few studies, although the low/resilient trajectory and the high/chronic trajectory were identified, the proportion of survivors following these two patterns are different compared to the majority. Two studies on breast cancer survivors reported that 75.5% and 38% of the survivors experienced chronically high FCR in 12 months and depression in 60 days, respectively [35, 40]. Similarly, one study on gynecological cancer also found that 38.5% of the cancer survivors experienced persistent high general distress throughout the chemotherapy [42].

Several dynamic patterns were also commonly observed. The improvement trajectory characterized by initially elevated distress that decreased over time and the worsening trajectory characterized by initially low distress that increased over time were identified in 12 studies and 10 studies, respectively. The proportion of cancer survivors following these patterns showed heterogeneous results, but in general, survivors were more likely to experience improved psychological distress during the follow‐up period (Supporting Information S1: Table S2).

### 3.6 Association Between Distress Change Patterns and Survival

The longitudinal changes of psychological distress over time, rather than single‐point assessments, provided prognostic information across studies. Survivors with persistent or worsening depressive symptoms consistently demonstrated worse survival outcomes in most studies. Head and neck cancer survivors with persistent/recurrent/late depressive symptoms had a 104% increased risk of mortality compared to those who never experienced depression [19], while hematological survivors with persistent depressive symptoms had a 117% increased mortality after adjustment [18]. One exception that focused on breast cancer survivors with an ICD diagnosis of depression, reported that no difference in the risk of dying was found during 2 years of follow‐up in the persistent depressive symptom group when compared to the never depressed group [21].

Survivors with new‐onset or emerging psychological distress also showed an elevated mortality risk. New‐onset depression carried a 50% increased mortality risk, while persistent depression showed a 42% increase [25]. Similarly, the emerging depressive symptom trajectory was found to be associated with the highest mortality risk among the four identified patterns, with almost 7‐fold increased risk compared to the no/resilient group (OR = 6.89) [24]. Notably, depression at the 24‐month assessment emerged as the strongest independent prognostic factor (HR: 1.992), suggesting that psychological status later in the cancer journey may have stronger prognostic significance than immediate post‐diagnosis distress [26].

Recovery from psychological distress was generally associated with improved survival outcomes. Survivors who recovered from initial depressive symptoms showed more favorable outcomes than those with persistent symptoms. No significant increase in mortality risk was found for survivors who recovered from depressive symptoms (HR = 1.12; 95% CI, 0.66–1.90) compared to those who never experienced depression [19], and survivors whose depression remitted had survival rates similar to those who never experienced depression (HR: 1.02, 95% CI: 0.79–1.31) [25]. However, two other studies reported that recovery was associated with a significant increase in risk of dying compared to the resilient group, possibly due to a more advanced disease stage at baseline [18, 24].

Among studies that examined continuous trends without trajectory categorization, a universal decrease in overall survival was found with worsening psychological symptoms [16, 17]. However, some studies showed mixed findings: only recurrence‐free survival, not overall survival, was associated with depressive symptoms [20], while in other studies, the univariate association became non‐significant in the multivariate analysis [22, 23].

### 3.7 Impact of Follow‐Up Duration on Survival Associations With Distress Patterns

The duration of follow‐up from psychological distress assessment to survival outcome revealed important temporal patterns in the prognostic significance of psychological distress changes. A study with a very short assessment time interval (preceding and following treatment) and also a short follow‐up period to survival outcome (median follow‐up time = 13.5 months) demonstrated immediate prognostic effects but primarily for non‐fatal outcomes. Depression symptom changes within the first month were associated with treatment response and progression‐free survival, though not overall survival, in advanced gastrointestinal cancer survivors [20]. Studies with intermediate follow‐up periods (1–5 years) showed the most consistent mortality associations, with HR clustering around 2 for persistent depression [18, 19, 21, 26], suggesting that psychological distress effects on survival become clinically apparent within this time frame for different cancer types. Studies with more than 5 years of follow‐up to survival outcome revealed highly heterogeneous findings. Effect sizes varied dramatically: from non‐significant associations after adjustment [22], to moderate associations (HR = 1.42–1.50) [25], to extremely large effects (OR = 6.89) [24]. A “time‐delayed effect” was identified where survival curves between high‐depressed and non‐depressed survivors diverged substantially only at 15–25 months post‐diagnosis, suggesting latency in prognostic manifestation [17].

### 3.8 Other Factors Influencing Distress Patterns and Their Impact on Survival

#### 3.8.1 Demographic Factors

Several demographic factors were consistently associated with distress trajectories across studies. Specifically, younger age was frequently associated with higher anxiety, depression, and FCR and overall distress [9, 27, 31, 34, 35, 39, 43, 50, 52]. Age also showed particularly notable effects on the relationship between psychological distress and survival. Older cancer survivors consistently demonstrated stronger associations between psychological distress and mortality risk. The association between depression and increased risk of death was particularly pronounced among breast cancer survivors aged 75 and older (HR: 2.67, 95% CI: 2.00–3.56) and those between 65 and 74 years (HR: 1.39, 95% CI: 1.04–1.84), while no significant increased risk was found in middle‐aged survivors (50–65 years) [21]. The hazard ratio for mortality associated with persistent depression was substantially higher among survivors over 65 (HR: 1.45, 95% CI: 1.28–1.65) compared to those under 65 (HR: 1.22, 95% CI: 1.05–1.42) [24]. A pronounced age gradient in mortality risk was revealed within the older adult group, with survivors over 80 showing substantially higher risk of death compared to those under 70 (HR: 3.07, 95% CI: 2.54–3.71), and those between 70 and 80 years demonstrating an intermediate risk (HR: 1.52, 95% CI: 1.29–1.80) [22]. Studies focusing exclusively on older cancer survivors provided further insights. Among cancer survivors over 70 years, higher depressive symptom scores were associated with increased mortality risk (HR = 1.16, 95% CI: 1.12–1.21) even after controlling for cancer‐related prognostic factors [16], and among older survivors with bladder cancer, changes in mental health had prognostic significance in this cohort, with better role, emotional, and social functioning associated with longer survival [23].

Female gender was generally associated with higher levels of psychological distress, particularly anxiety and FCR and overall distress [27, 31, 44, 50, 51, 53]. Female gender showed mixed associations in prognostic significance—protective in some studies [16] but associated with higher psychological distress risk in others [26]. Lower socioeconomic status, educational level, unemployment, and unmarried status were also associated with higher risk of persistent or worsening depression trajectories in multiple studies [9, 21, 29, 31, 33, 34, 36, 43, 50, 51, 55], and consistently predicted worse survival outcomes [19, 26].

#### 3.8.2 Clinical Factors

The evolution of distress and prognostic significance also varied by cancer type and cancer stage [25, 29, 37, 50, 53]. Time effects were obtained in survivors with breast, prostate, and gynecological cancer [37]. The association between distress and reduced survival was more pronounced among lung cancer survivors (HR: 1.79, 95% CI: 1.42–2.25) compared to other cancer types [25]. Depression symptoms were also associated with increased mortality among breast and lung cancer survivors with early‐stage cancer (stages I and II) [21, 25]. Conversely, in colorectal cancer, late‐stage disease emerged as a significant predictor of an unfavorable distress trajectory where survivors were more vulnerable to higher distress [33, 50]. Head and neck cancer studies revealed that the association between depressive symptoms and survival was stronger in HPV‐negative compared to HPV‐positive oropharyngeal cancer survivors [19].

#### 3.8.3 Psychosocial Resources

Several studies investigated the role of psychosocial resources in modifying distress trajectories and their impact on survival. Higher social support was associated with more favorable depression trajectories [35], and the negative impact of deteriorating emotional well‐being on survival was attenuated among survivors reporting high levels of social support [23]. Coping strategies also emerged as important factors, with avoidant coping being associated with worsening anxiety and depression trajectories, while more adaptive coping strategies were associated with decreasing FCR over time [43, 53]. While psychosocial resources like sense of control showed prognostic significance in univariate analyses [17], these effects were generally attenuated in multivariate models including psychological distress measures.

## 4 Discussion

This scoping review synthesized evidence from 41 longitudinal studies on psychological distress changes over time, with 11 of them examining the relationship between changes in psychological distress and survival outcomes in cancer survivors. The findings consistently demonstrate that the trajectory of psychological distress over time, rather than single‐point assessments, provides prognostic information for cancer survival. Across all studies, the majority of cancer survivors maintained low psychological distress throughout their cancer journey, while a minority experienced chronically elevated symptoms that showed minimal improvement over time. Across diverse cancer populations, survivors with persistent or worsening psychological distress showed significantly worse survival outcomes, while those who recovered from initial symptoms demonstrated survival rates comparable to survivors who never experienced psychological distress.

The studies included in this review demonstrated overall high quality and several methodological strengths, including prospective longitudinal designs, validated psychological assessment instruments, and appropriate statistical methods for handling time‐dependent variables. Longitudinal assessment of distress offers an additional perspective on changes over time, complementing cross‐sectional measures, and may help identify subgroups with emerging or worsening symptoms that could have prognostic implications. The consistency of findings across diverse cancer populations and different geographic settings strengthens the generalizability of the results. Additionally, this scoping review provides a comprehensive synthesis of evidence from multiple cancer types, ranging from common cancers like breast and colorectal to rarer malignancies, enhancing the breadth of applicability. The review also examined a unique aspect of the literature by analyzing temporal patterns across different follow‐up durations, revealing that the prognostic significance of psychological distress may become more relevant with longer follow‐up. Different follow‐up periods may reveal distinct aspects of the distress‐survival relationship not captured in studies with short follow‐up.

### 4.1 Interaction Between Distress Constructs

Several studies revealed complex interactions between anxiety, depression, and fear of cancer recurrence, suggesting these psychological constructs may follow distinct patterns and relationships. Anxiety and depression demonstrated different age‐related patterns, with older survivors showing lower anxiety about cancer compared to younger survivors, while paradoxically exhibiting higher depression levels [9]. Additionally, significant associations were identified between depression and moderate levels of FCR, suggesting that these psychological constructs may influence each other in complex ways [55]. These interactions highlight the importance of examining multiple psychological dimensions simultaneously rather than treating them as independent constructs, as their interplay may have different implications for both psychological adaptation and survival outcomes in cancer survivors.

### 4.2 Potential Mechanisms Linking Distress and Survival

Several potential mechanisms may explain the observed relationship between psychological distress change patterns or trajectories and cancer survival. Physiological pathways include the impact of chronic stress on immune function, with persistent psychological distress potentially leading to downregulation of natural killer cell activity and other immune responses critical for cancer surveillance [16]. Particularly intriguing is the consistent finding that psychological distress had stronger associations with survival in early‐stage disease compared to advanced disease, as demonstrated in both breast cancer [21] and lung cancer [25]. This stage‐dependent effect may reflect the greater influence of psychosocial factors on survival when biological prognostic factors are less deterministic. In advanced disease, tumor biology may overwhelm any potential influence of psychological factors, whereas in early‐stage disease, psychological status may interact more meaningfully with immune function, treatment adherence, or other pathways that influence disease progression. The observation that health‐related quality of life changes predicted survival [23] may reflect underlying biological processes, as declining physical function could represent early indicators of disease progression that precede clinically apparent recurrence. Additionally, psychological distress may influence treatment adherence, with depressed survivors potentially less likely to complete planned treatment regimens or seek timely medical care when complications arise. Depression was found to be associated with decreased receipt of guideline‐recommended treatment, suggesting that behavioral pathways may partially mediate the relationship between psychological distress and survival [21]. Several cohort studies and reviews report that patients with pre‐existing mental disorders are less likely to receive guideline‐concordant cancer treatment; a few mediation analyses suggest that later stage at diagnosis explains a small proportion of the excess mortality, whereas treatment‐timing (delay) does not consistently mediate the effect [56, 57, 58]. Furthermore, behavioral pathways (e.g., reduced adherence, ongoing smoking, inactivity) and biological mechanisms (e.g., systemic inflammation, impaired tumor response) have also been proposed and tested in some cancer‐psychiatric comorbidity studies, though evidence is heterogeneous and context‐specific [59, 60]. Behavioral mechanisms may also contribute to the observed associations. Survivors with persistent psychological distress may be more likely to engage in health‐compromising behaviors such as smoking, excessive alcohol consumption, or poor nutritional habits, as suggested when lifestyle factors were included in analyses [19]. The observation that adjusting for lifestyle factors attenuated but did not eliminate the association between psychological distress and survival suggests that both physiological and behavioral pathways may contribute to the observed relationship. Suicide was also discussed as a potential explanation for findings, since analysis of all‐cause mortality indicated that high psychological distress may contribute to increased suicide rates among cancer survivors [19]. However, the persistence of psychological factors as independent predictors after controlling for disease characteristics across multiple studies suggests that psychological mechanisms contribute meaningfully to cancer outcomes beyond their role as markers of disease severity.

### 4.3 Study Limitations

This scoping review has several limitations warrant consideration. Limiting our search to English‐language publications may have resulted in the exclusion of relevant literature from smaller studies that were only published in local language. The significant heterogeneity in sample characteristics across included studies makes drawing definitive conclusions about the impact of distress change patterns on survival challenging, as these factors are likely integral components of the complex relationship between psychological distress and clinical outcomes. Age‐related considerations present another limitation, as the expression of depressive symptoms in older survivors with cancer is complex, with greater heterogeneity than in younger counterparts [61]. Most studies in this review did not explicitly examine age‐stratified effects of psychological distress on survival, limiting understanding of whether interventions should be tailored by age group. Evidence regarding psychological distress and survival among middle‐aged and younger adults was particularly limited, with few studies specifically analyzing the relationship between distress trajectories and survival outcomes in younger survivors [17, 21]. Several methodological factors may influence interpretation of findings. The heterogeneity in psychological assessment instruments presents challenges for direct comparisons and may contribute to observed differences in effect sizes. Different instruments capture varying aspects and severity of psychological distress, potentially leading to inconsistent trajectory classifications. Several studies adjusted cut‐off values to account for geographic or ethnic population differences [18, 45] Some employed multiple correlated instruments, which may have resulted in psychological distress variables not emerging as independent predictors in multivariate analyses due to collinearity [22]. Similarly, the diversity in analytical approaches—ranging from simple change scores to sophisticated growth mixture modeling—may yield different conclusions regarding both trajectory prevalence and survival associations. A critical limitation is the predominant reliance on only two assessment time points for identifying change patterns among those examining distress‐survival associations, which may inadequately capture complex, non‐linear patterns of psychological adaptation throughout cancer survivorship. Studies with very long follow‐up periods experienced high dropout rates, potentially due to mortality, which may bias results. Given the substantial variability across studies in the timing of assessments, follow‐up durations, and the specific constructs used to operationalize psychological distress, the patterns identified in this review should be interpreted as thematic rather than as evidence of equivalence between syndromes or study designs. The aim of this scoping review was to map consistent directional trends across heterogeneous studies, rather than to assume uniformity or pool effects across differing constructs.

However, the main findings should not be interpreted as implying that psychological distress predicts or causes poorer survival. Rather, the evidence indicates that survivors who experience persistent, worsening, or new‐onset distress over time tend to have worse survival outcomes compared with those whose symptoms remain stable or recover. While it remains possible that confounders as underlying disease progression may influence both distress and survival, the majority of included studies accounted for potential confounders, such as key sociodemographic (age, sex), clinical and psychosocial factors (cancer stage, comorbidities, social support). Most studies found that distress trajectories remained significantly associated with poorer survival even after adjustment for these factors, supporting the interpretation that changes in psychological distress over time have independent prognostic significance. Nonetheless, given the observational nature of the studies and the limited number of assessment time points in some cohorts, the potential for reverse causation cannot be entirely excluded, especially in those studies with short follow up time to death outcome (< 3 years), as declining physical health or disease progression may lead to worsening psychological symptoms, rather than psychological distress independently influencing survival.

Despite these methodological challenges, the inclusion of studies with varying analytical approaches provides insight into the robustness of findings across different methodological frameworks. The consistency of findings across diverse populations, cancer types, and study designs provides compelling evidence for the prognostic significance of longitudinal psychological distress patterns.

### 4.4 Clinical Implications and Future Research Directions

The observation that recovery from initial psychological distress is associated with improved survival outcomes suggests that targeted interventions promoting psychological resilience may have important prognostic implications. Evidence indicates that resilience‐promoting programs for cancer survivors that are tailored to specific target groups have performed better in scientific studies than universal programs [62]. These findings highlight the urgent need for widespread implementation of screening protocols in routine care based on established clinical guidelines.

Although our review indicates that persistent or worsening psychological distress is linked to poorer prognosis, current evidence from intervention studies suggests that reducing distress does not necessarily translate into improved survival [63]. Future research should explore whether targeted psychosocial interventions can influence long‐term clinical outcomes, ideally in well‐powered trials with long follow‐up. Future research should consider a deeper investigation of the pathways through which psychological distress influences survival outcomes. Social resources represent a particularly important mediating factor that requires further examination. Research has identified multiple dimensions of social resources among long‐term cancer survivors, including social networks, social support, and personal resources such as family relationships, partnerships, optimism, self‐efficacy, and hope [64, 65, 66]. However, the evidence for a consistent effect of positive thinking on survival is mixed. Some critical reviews [67] suggest that emphasizing a positive mindset may not improve survival outcomes and could potentially add psychological burden to patients. These observations highlight the importance of focusing on interventions that enhance quality of life and emotional well‐being, rather than assuming that optimism alone can influence cancer prognosis. The clinical implication is the importance of routine distress screening and timely psychosocial support. Understanding how changes in psychological distress affect these social resources, and how these subsequently influence survival outcomes, could inform the development of more effective interventions. Additionally, very limited studies have examined interactions among multiple psychosocial factors and their combined effects on survival outcomes. Future research should explore how different psychological trajectories (depression, anxiety, FCR) interact with social resources and other resilience factors to influence long‐term health outcomes. This approach could facilitate the development of comprehensive risk stratification models to identify individuals most susceptible to persistent distress and poor survival outcomes. Lastly, the few long‐term follow‐up studies [17, 23] indicate a potentially cumulative effect of psychological distress on long‐term cancer outcomes, emphasizing the importance of very‐long‐term follow up.

## 5 Conclusion

This scoping review synthesized evidence from longitudinal studies suggesting that the trajectories of psychological distress may be useful for predicting outcomes in cancer survivors. While persistent or worsening distress was generally associated with worse survival outcomes, the effect sizes varied considerably across studies and findings were not uniformly consistent, particularly among survivors who experienced symptom remission.

These findings support a shift beyond single time‐point assessments toward longitudinal monitoring of distress in both research and clinical contexts. Whether modifying distress trajectories through psychosocial interventions can improve survival outcomes remains unclear and warrants further investigation through well‐designed intervention studies. In the interim, incorporating routine longitudinal distress assessment into survivorship care using tailored supportive approaches may provide additional prognostic insight.

## Author Contributions

**Yifeng Gao:** conceptualization, methodology, data curation, investigation, formal analysis, visualization, writing – original draft, writing – review and editing. **Chien‐Tzu Lee:** data curation. **Daniela Doege:** conceptualization, methodology, writing – review and editing, supervision. **Melissa S. Y. Thong:** conceptualization, writing – review and editing, supervision. **Volker Arndt:** conceptualization, writing – review and editing, supervision.

## Funding

Y.G. was supported by a doctoral stipend from the German Cancer Research Center (Deutsches Krebsforschungszentrum, DKFZ). C.‐T.L. was supported by a scholarship from the German Academic Exchange Service (Deutscher Akademischer Austauschdienst, DAAD). The funders had no role in the study design, data collection and analysis, decision to publish, or preparation of the manuscript.

## Ethics Statement

All data analyzed for this study were extracted from published reports of previously conducted studies. No primary data collection occurred; therefore, this study was not subject to IRB review.

## Conflicts of Interest

The authors declare no conflicts of interest.

## Supporting information


Supporting Information S1

## Data Availability

Data sharing not applicable to this article as no datasets were generated or analyzed during the current study.
