# Supplementary material for: Development of Psychosocial Distress in Cancer Survivors and Its Potential Prognostic Impact on Survival: A Scoping Review
Source: Psychooncology. 2026 May 15;35:e70488. doi: 10.1002/pon.70488 (PMC13178206; doi:10.1002/pon.70488)
Supplement: Supplementary file 1 — Supporting Information S1 [file PON-35-e70488-s001.docx]

**Full search strategies**

Pubmed:

(((psychological OR psychosocial OR mental OR emotional) AND (distress* OR "well-being")) OR depress* OR anxi* OR "fear of recurrence" OR "fear of progression" OR "Stress, Psychological"[MeSH Terms] OR "Depression"[MeSH Terms] OR "Anxiety"[MeSH Terms] OR "Mental Health"[MeSH Terms])

**AND** (((cancer OR neoplasm OR "malignant tumor") AND (survivor* OR patient*)) OR Neoplasms*[MeSH Terms] OR Cancer Survivors[MeSH Terms])

**AND** (mortality OR death* OR prognos* OR surviv* OR Prognosis[MeSH Terms] OR Mortality[MeSH Terms])

**AND** ("cohort stud*" OR population-based OR "Cohort Studies"[MeSH Terms] OR "Longitudinal Studies"[MeSH Terms] OR "Follow-Up Studies"[MeSH Terms])

**NOT** (childhood OR adolescent*)

**NOT** ("cross-sectional" OR "randomized controlled trial")

Web of Science:

TS=(((psychological OR psychosocial OR mental OR emotional) AND (distress* OR "well-being")) OR depress* OR anxi* OR "fear of recurrence" OR "fear of progression")

**AND** TS=((cancer OR neoplasm OR "malignant tumor") AND (survivor* OR patient*))

**AND** TS=(mortality OR death* OR prognos* OR surviv*)

**AND** TS=("cohort stud*" OR population-based OR "longitudinal stud*" OR "follow-up stud*")

**NOT** TS=(childhood OR adolescent*)

**NOT** TS=("cross-sectional" OR "randomized controlled trial")

PsycInfo:

(((psychological OR psychosocial OR mental OR emotional) AND (distress* OR "well-being")) OR depress* OR anxi* OR "fear of recurrence" OR "fear of progression" OR DE "Psychological Stress" OR DE "Depression" OR DE "Anxiety" OR DE "Mental Health")

**AND** (((cancer OR neoplasm OR "malignant tumor") AND (survivor* OR patient*)) OR DE "Neoplasms" OR DE "Cancer Survivors")

**AND** (mortality OR death* OR prognos* OR surviv* OR DE "Prognosis" OR DE "Mortality")

**AND** ("cohort stud*" OR population-based OR DE "Cohort Studies" OR DE "Longitudinal Studies" OR DE "Follow-Up Studies")

**NOT** (childhood OR adolescent*)

**NOT** ("cross-sectional" OR "randomized controlled trial")

| **Table S1**. Quality assessment of 30 studies on longitudinal psychological distress trajectory by modified Newcastle-Ottawa quality assessment scale (NOS) ^†^ for cohort studies | | | | | | | | |
| --- | --- | --- | --- | --- | --- | --- | --- | --- |
| **Study** | **Selection**  **(3 stars)** | | | **Comparability**  **(2 stars)** | **Outcome**  **(3 stars)** | | | **Modified**  **final score (8 stars)** |
|  | Representativeness of the exposed cohort | Ascertainment of exposure | Outcome of interest was not present at start of study | Comparability of cohorts on the basis of the design or analysis^‡^ | Assessment of outcome | Was follow-up long enough for outcomes to occur | Adequacy of follow up of cohorts |  |
| Avis et al., 2020 | * | * | * | ** | * | * | * | 8 |
| Beesley et al., 2020 | * | * | * | ** | * | * | * | 8 |
| Bidstrup et al., 2015 |  | * | * | ** | * | * | * | 7 |
| S.L. Brown et al., 2023 | * | * | * | * | * | * | * | 7 |
| Charles et al., 2022 | * | * | * | ** | * | * | * | 8 |
| Deuning-Smit et al., 2022 | * | * | * | ** | * | * | * | 8 |
| J. Dunn et al., 2013 | * | * | * | ** | * | * | * | 8 |
| L.B. Dunn et al., 2011 | * | * | * | * | * | * | * | 7 |
| Ernster et al., 2023 |  | * | * | * | * | * |  | 5 |
| Halkett et al., 2022 |  | * | * | ** | * | * |  | 6 |
| Kant et al., 2018 |  | * | * | ** | * | * | * | 7 |
| Kwon et al., 2023 | * | * | * | ** | * | * | * | 8 |
| Lacourt et al., 2022 |  | * | * | * | * | * | * | 6 |
| Lam et al., 2013 | * | * | * | ** | * | * | * | 8 |
| Li et al., 2024 |  | * | * | ** | * | * | * | 7 |
| Liu and Wang., 2022 |  | * | * | * | * | * | * | 6 |
| Liu et al., 2022 | * | * | * | * | * | * | * | 7 |
| McGinty et al., 2016 |  | * | * | * | * | * | * | 6 |
| Mielcarek et al., 2016 |  | * | * | * | * | * | * | 6 |
| Mols et al., 2018 | * | * | * | ** | * | * | * | 8 |
| Pu et al., 2024 | * | * | * | ** | * | * | * | 8 |
| Qaderi et al., 2021 | * | * | * | * | * | * | * | 7 |
| Rottmann et al., 2016 | * | * | * | ** | * | * | * | 8 |
| Saboonchi et al., 2015 | * | * | * | * | * | * | * | 7 |
| Savard and Ivers, 2013 | * | * | * | ** | * | * | * | 8 |
| Séguin Leclair et al., 2019 | * | * | * | * | * | * | * | 7 |
| Shim et al., 2020 |  | * | * | ** | * | * | * | 7 |
| Stanton et al., 2015 |  | * | * | * | * | * | * | 6 |
| Wang et al., 2024 |  | * | * | ** | * | * | * | 7 |
| Yang et al., 2024 |  | * | * | ** | * | * | * | 7 |
| ^†^The standard NOS (max 9 stars) assesses selection, comparability, and exposure/outcome. For studies on longitudinal distress in cancer survivor cohorts, a non-exposed group was not required; therefore, a modified NOS (max 8 stars) was applied, excluding this criterion. ^‡^This section consists of 2 questions accounting for 1 star per question: “Study controls for most important factor” and “Study controls for additional important factor”. | | | | | | | | |

| **Table S2.** Characteristics of 30 included studies on longitudinal psychological distress trajectory in cancer survivors | | | | | | | | | | |
| --- | --- | --- | --- | --- | --- | --- | --- | --- | --- | --- |
| **Main author,  year,  country** | **Design** | **Data source** | **Cancer type** | **Population**^†^ | **Distress instrument**  **(cut-off values)** | **Sample size**^‡^ | **Assessment** | **Follow-up Length**^§^ | **Modelling** | **Result** |
| Avis et al.  2020  USA | Retrospective cohort | The Study of Women's Health Across the Nation (SWAN) Breast Cancer Survivors study | Breast | - Mean age: 56.5 ± 6.2 years - Disease stages: 0 to III | Mental well-being:  SF-36 MCS (continuous) | 141 | 6:  At SWAN FU 6; At FU 8; At FU 10;  At FU 12; At FU 13; At FU 15 | 10 years | Group-based trajectory modelling (GBTM) | 2 trajectories mental well-being: - consistently good MCS (88.4%) - very low MCS (11.6%) |
| Beesley et al. 2020,  Australia | Prospective cohort | The Primary Project in Queensland | Melanoma | - Mean age: 62 ± 14 years - Female: 42% - Tumor stages: 1b to 4b | Anxiety and Depression:  HADS-A and HADS-D (“subclinical” = 8-10; “clinical” = 11-21) | 675 | 7: At baseline;  6 months;  12 months;  18 months;  24 months;  36 months;  48 months | 4 years | GBTM | 4 trajectories for anxiety:  - persistent clinical symptoms (1%) - clinical symptoms decreasing to normal (6%) - clinical symptoms decreasing to sub-clinical (10%) - normal stable (84%) 4 trajectories for depression:  - persistent symptoms (3%) - symptoms decreasing to normal (6%) - normal increasing to borderline symptoms (8%) - normal stable (84%) |
| Bidstrup et al. 2015 Denmark | Retrospective cohort | The Breast Surgery Clinic of Copenhagen University Hospital | Breast | - Mean age: 61 (28-88) years | Overall distress, anxiety and depression:  DT (cut-off ≥ 7), HADS-A and HADS-D (“doubtful cases” = 8-10; “cases” = 11-21) | 323 | 3: Before surgery; 4 months; 8 months | 8 months | TRAJ finite mixture model procedure | 5 trajectories for overall distress: persistent severe distress (8%) 3 trajectories for anxiety: moved from severe anxiety to moderate below the cut-off (76%) 4 trajectories for depression: continuously moderately severe symptoms (67%) |
| S.L. Brown et al. 2023  UK | Retrospective cohort | Liverpool Ocular Oncology Centre | Uveal melanoma | - Mean age: 69.37 ± 12.39 years - Female: 48.8% | FCR, anxiety and depression (overall distress):  FCR scale (continuous), HADS (cut-off ≥ 7) | 475 | 8: 6-month posttreatment; 12-month; 24-month;  36-month;  48-month; 60-month;  72-month;  84-month | 7 years | Growth mixture modelling (GMM) | 2 trajectories for anxiety: - consistently low (82.5%) - consistently elevated (17.5%) 2 trajectories for depression: - consistently low (89.1%) - consistently elevated (10.9%) 2 trajectories for FCR: - consistently low (80.6%) - consistently elevated (19.4%) |
| Charles et al. 2022  France | Retrospective cohort | The Deeper in the Understanding and Prevention of Depression in Breast Cancer Patients cohort study | Breast | - Mean age: 56.2 ± 11.2 years - Disease stages: I to III | Depression:  HADS-D (“doubtful cases”: 8-10; “probable cases” ≥ 11) | 4,803 | 4: At diagnosis (T0); 3 to 6 months after treatment (T1); 12 months after treatment (T2);  36 months after treatment (T3) | 3 years | GBTM | 6 trajectories for depression: - non-cases with no expression of symptoms (54.8%) - intermediate worsening (22.4%) - intermediate improvement (10.0%) - remission (5.4%) - delayed occurrence (4.2%) - stable depression (3.2%) |
|  |  |  |  |  |  |  |  |  |  |  |
| Deuning-Smit et al. 2022  the Netherlands | Prospective cohort | 5 oncological centers and 3 general hospitals - the NET-QUBIC cohort study | Head and neck | - Mean age: 63.5 ± 9.4 - Female: 25.9% - Disease stages: 0 to IV | FCR:  CWS-6  (high ≥ 12) | 617 | 3: At diagnosis; 3 months post-treatment; 6 months post-treatment | 6 months | Latent Class Growth Analysis (LCGA) | FCR decreased slightly between baseline and 3 months post-treatment and remained stable up to 6 months;  2 trajectories for FCR:  - high stable (n = 125)  - low declining (n = 492) |
| J. Dunn et al. 2013  Australia | Retrospective cohort | The Quessland cancer registry | Colorectal | - Age: 20-80 years - Female: 39.9% | Overall distress:  BSI-18 GSI (“case” ≥ 50) | 1,706 | 6: 5 months after diagnosis (T1); 12 months (T2);  24 months (T3);  36 months (T4); 48 months (T5); 60 months (T6) | 5 years | GMM | 4 trajectories for overall distress:  - constant low (19.4%) - medium level (29.6%) - medium increase (38.5%)  - high decrease (12.5%) |
| L.B. Dunn et al. 2011  USA | Retrospective cohort | 1 Cancer Center, 2 public hospitals, and 4 community practices | Breast | - Mean age: 54.9 ± 11.6 years - Disease stages: 0 to IV | Depression:  CES-D (“clinical” ≥ 16) Anxiety: STAI-T (“high” ≥ 31.8) AND STAI-S (“high” ≥ 32.2) | 398 | 7: Prior to surgery 1 month after surgery 2 months; 3 months; 4 months; 5 months; 6 months | 6 months | GMM | 4 trajectories for depression:  - resilient (38.9%) - subsyndromal (45.2%) - delayed (11.3%) - peak (4.5%) |
| Ernster et al. 2023 USA | Prospective cohort | The UF Health Neuro-Oncology Clinic | Brain | - Age: 18-95 years - WHO tumor grades: I to IV | Anxiety and depression:  PROMIS anxiety and depression short forms (“normal” < 55; “mild” = 55 to 55.9; “moderate” = 60 to 60.9; “severe” > 70) | 74 | A minimum 8-week interval between assessments | 18 months | Linear mixed-effects models; LCGA | No significant changes in anxiety or depression scores over time;  5 trajectories for anxiety: - decreased from moderate to mild (n=3) - decreased but still mild (n=24) - decreased within the normal range (n=7) - increased from normal to mild (n=6) - increased within the normal range (n=8) 5 trajectories for depression: - decreased but still clinically elevated (n=1) - increased from mild to moderate (n=9) - increased from normal to mild (n=12) - increased within the normal range (n=12) - decreased from normal to the lowest (n=14) |
| Halkett et al. 2022  Australia | Prospective cohort | 4 tertiary hospitals in 2 Australian states | High grade glioma (brain) | - Mean age: 55.7 ± 13.0 - Female: 29% - WHO tumor grades: III to IV | Overall distress:  DT (“moderate” ≥ 4; “severe” ≥ 7) | 116 | 3: During chemoradiotherapy; 3 months later; 6 months later | 6 months | GBTM | 4 trajectories for overall distress:  - consistent low (18%) - low to high (38%) - high to low (24%) - consistent high (19%) |
| Kant et al. 2018  Germany | Prospective cohort | 2 breast cancer centers | Breast | - Mean age: 52.96 ± 9.85 years | Overall distress:  12- items GHQ (cut-off = 11/12) | 181 | 4: After surgery/biopsy; At treatment completion; 2 months thereafter; 6 months thereafter | 6 months | Latent growth mixture modelling (LGMM) | 4 trajectories for overall distress:  - resilient (73.1%) - high-remitting (7.7%) - delayed increase (7.9%) - high chronic (11.3%) |
| Kwon et al. 2023  Canada | Retrospective cohort | The Ontario Cancer Registry linked with administrative health data | Mixed | - Mean age: 64.5 ± 13.4 years Female: 51.7% | Overall distress:  depression, anxiety, and well-being items of ESAS-r (continuous) | 3,416 | 7: Within 1 month of initial consultation since diagnosis; 1 to < 2 months since consultation; 2 to < 3 months; 3 to < 4 months; 4 to < 5 months;  5 to < 6 months; 6 to < 7 months | 6-7 months | LGMM | 4 trajectories for anxiety, depression and well-being:  - moderate symptoms with slight improvement (21%) - minimal to moderate symptoms (29%) - severe symptoms with substantial improvement (9%) - minimal symptoms remaining stable (40%) |
| Lacourt et al. 2022  USA | Retrospective chart review | The University of Texas MD Anderson Cancer Center | Breast | - Mean age: 49.4 ± 11.83 years - Disease stages: I to IIIC | Overall distress:  DT (“low” < 4; “significant” = 4-6; “high” ≥ 7) | 252 | 13: The month prior to chemo onset;  At least monthly for the 12 months following chemo onset | Mean: 8 months | Growth mixture modelling (GMM) | Distress showed a cubic growth trajectory with highest distress prior to treatment onset followed by a steep decline in the first three months of treatment. A slight increase in distress was apparent over months 6–10 |
| Lam et al. 2013  Hong Kong | Prospective cohort | 6 public hospital breast center/ oncology out-patient clinics | Breast | - Mean age: 53.51 ± 9.84 years - Disease stages: III to IV | Anxiety and Depression:  HADS-A and HADS-D (“non-cases” < 8; “subclinical” = 8-10; “clinical” > 10) | 192 | 5: Before first chemo; At 1.5 months thereafter; 3 months thereafter; 6 months thereafter; 12 months thereafter | 12 months | LGMM | 4 trajectories for anxiety:  - low-stable (68.2%) - delayed (13.0%) - recovery (9.9%) - high-stable (8.9%) 4 trajectories for depression:  - low-stable (67.7%) - recovering (18.8%) - high-stable (9.4%) - high-recovering (4.1%) |
| Li et al. 2024  China | Retrospective cohort | Convenience sample in a general hospital | Gynecological: cervical, ovarian, endometrial | - Mean age: 55.46 ± 11.12 years - Disease stages: I to IV | Overall distress:  DT and Problem List (continuous) | 132 | 4: 1 day before surgery; Before the first chemotherapy;  At the third chemotherapy;  End of the last chemotherapy | unknown: throughout surgery and chemotherapy | latent class growth model (LCGM) | 3 trajectories for overall distress:  - high level decline (37.4%) - no psychological distress (24.1%) - high level stable (38.5%) |
| Y.J. Liu and Wang 2022 China | Prospective cohort | Heilongjiang Provincial Hosiptal | Gastric | - Mean age: 58.0 ± 11.5 years - Female: 37.2% - TNM stages: I to III | Anxiety and depression:  HADS-A and HADS-D (“mild” = 8-10; “moderate” = 11-14; “severe” = 15-21) | 226 | 4:  At baseline (M0); 12th month after hospital discharge; 24th month;  36th month (M36) | 36 months | Repeated measures ANOVA | HADS-A and HADS-D scores were gradually increased from M0 to M36, and their occurrences and grades were also worsened piece by piece |
| Liu et al. 2022  Sweden | Prospective cohort | The nationwide Oesophageal Surgery on Cancer Patients-Adaption and Recovery (OSCAR) study | Esophageal | - Mean age: 66.3 ± 8.5 years - Female: 14.6% - Tumor stages: I to IV | Overall distress:  HADS-A or HADS-D (“possible-probable” ≥ 8) | 192 | 3:  1-year post-surgery; 1.5 years; 2 years | 2 years | Latent growth curve model (LGCM) | There was no statistically significant variation in this longitudinal growth trajectory of psychological distress, whereas substantial individual differences were found in the probability of reporting psychological distress at 1 year after surgery |
| McGinty et al. 2016  USA | Retrospective chart review | Moffitt Cancer Center | Breast | - Mean age: 61.48 ± 9.60 years - Disease stages: 0 to IIIA | FCR: VASs;  CWS (continuous) | 161 | 6: 1 month prior to the mammogram; 1 week prior to the mammogram; Immediately prior to the mammogram; Immediately following the results; 1 week after the results; 1 month after the results | 60 days | Growth curve analysis; GMM; repeated-measures ANOVA | FCR significantly changed over time with scores increased prior to the mammogram, decreased immediately following receipt of negative mammography results, and increased during the month following the mammogram;  2 trajectories for FCR:  - higher FCR (75.5%) - lower FCR (24.5%) |
| Mielcarek et al. 2016  Poland | Retrospective cohort | The Department of Gynecology Medical University of Gdansk | Ovarian | - Mean age: 53.9 ± 10.8 - Disease stage: advanced | Depression and Anxiety:  HADS-A, HADS-D (“non-case” = 0-7; “possible case” = 8-10; “probable case” = 11-21) and STAI (continuous) | 106 | 4:  Prior to surgery; 1-week after surgery; Before the 2nd course of Chemotherapy;  Before the 4th course of Chemotherapy | Mean: 112 days | Repeated-measures ANOVA | Either the STAI trait or HADS depression level remained relatively stable across the therapeutic process |
| Mols et al. 2018  the Netherlands | Retrospective cohort | The Patient-Reported Outcomes Following Initial Treatment and Long-Term Evaluation of Survivorship (PROFILES) Registry | Colorectal | - Mean age: 69.4 ± 9.5 - Female: 44.9% - Disease stages: I to IV | Anxiety and depression:  HADS-A and HADS-D (cut-off ≥ 8) | 2,625 | 4:  2010; 2011;  2012;  2013 | 4 years | Linear mixed effects models | Overall trend: Anxiety was stable, whereas depression scores changed over time;  3 trajectories for depression: - continuously high (8.3%) - fluctuating (21.1%) - continuously low (70.6%) 3 trajectories for anxiety: - continuously high (9.6%) - fluctuating (22.3%) - continuously low (68.1%) |
| Pu et al. 2024  USA | Retrospective cohort | The National Health and Aging Trends Study (NHATS) | Mixed | - Age: ≥ 65 years - Female: 52.38% | Anxiety and depression (overall distress):  PHQ-4 (cut-off ≥ 3) | 1,766 | 7:  Annually between 2015 and 2021 | 6 years | GBTM | 4 trajectories for overall distress:  - sustained low risk (48.9%) - deteriorating (24.1%) - meliorating (11.0%) - sustained high risk (16.1%) |
|  |  |  |  |  |  |  |  |  |  |  |
| Qaderi et al. 2021 the Netherlands | Retrospective cohort | The Prospective Dutch Cohort CRC (PLCRC) | Colorectal | - Mean age: 65.5 ± 9.6 years for colon cancer; 64.4 ± 9.7 years for rectal cancer - Female: 38% for colon cancer; 31% for rectal cancer - Disease stages: I to III | Overall distress:  HADS (cut-off ≥ 11) | 1,535 | 6:  At study enrolment; 3 months; 6 months; 12 months; 18 months; 24 months | 24 months | LCGA | 3 trajectories for overall distress:  - low distress (64.0%) - moderate distress (26.9%) - high distress (9.1%) |
| Rottmann et al. 2016 Denmark | Retrospective cohort | The Danish Couples and Breast Cancer Cohort (DCBCC) | Breast | - Mean age: 58.2 ± 10.4 years | Depression:  CES-D (“clinical meaningful” ≥ 16) | 546 | 3:  The phases of active treatment; 5 months thereafter; 12 months; | 12 months | Trajectory finite mixture model | 3 trajectories for depression:  - high stable (13%) - intermediate decreasing (38%) - low (49%) |
| Saboonchi et al. 2015 Sweden | Prospective cohort | 3 clinics in Stockholm | Breast | - Mean age: 51.2 ± 8.2 years | Anxiety:  HADS-A (continuous) | 725 | 4:  4 weeks after surgery;  12 months;  18 months; 24 months | 24 months | Latent Growth Modelling (LGM) and GMM | 4 trajectories for anxiety:  - high stable (6.2%) - high decrease (15.6%) - mid decrease (33%) - low decrease (45%) |
| Savard and Ivers 2013 Canada | Prospective cohort | L'Hotel-Dieu de Quebec (CHUQ) and Hospital du St-Sacrement (CHA) | Mixed | - Mean age: 57.0 ± years - Female: 64.4% - Cancer stages: 0 to IV | FCR:  Severity Subscale of FCRI (“clinical” ≥ 13) | 962 | 6:  Baseline (T1); 2 months (T2);  6 months;  10 months;  14 months; 18 months | 18 months | Linear and generalized mixed models | An overall significant time effect was found, with a significant reduction from T1 to T2 and a stable trend throughout the remainder of the study |
| Séguin Leclair et al. 2019 USA | Prospective cohort | The American Cancer Society's Study of Cancer Survivors-I (SCS-I) | Mixed | - Mean age: 56.22 ± 11.19 years - Female: 60.4% - Cancer stages: 0 to III | FCR:  CIPLS-FCR subscale (continuous) | 2,337 | 3:  Mean 1.3 years postdiagnosis;  Mean 2.2 years; Mean 8.8 years | 9 years | Mixture model; repeated-measures ANOVA | 3 trajectories for FCR:  - low (33.6%) - moderate (58.1%) - high (8.3%) |
| Shim et al. 2020 South Korea | Prospective cohort | Asian Medical Center in Seoul | Breast | - Mean age: 47.90 ± 9.17 years - Disease stage: 0 to III | FCR:  FOP-SF (continuous) | 162 | 5:  Baseline; 3 months following surgery;  6 months;  12 months;  18 months | 18 months | LCGA | 3 trajectories for FCR:  - low decreasing (38.31%) - moderate decreasing (39.89%) - high (21.80%) |
| Stanton et al. 2015 USA | Prospective cohort | 2 oncology clinics in the greater Los Angeles area and at the University of Arizona Cancer Center (Tucson) | Breast | - Mean age: 56.4 ± 12.6 years - Cancer stages: 1 to 4 | Depression:  CES-D (cut-off ≥ 16) | 460 | 7  Within 4 months after diagnosis (study entry); Every 6 weeks for 6 months after study entry (4 times); 9 months after study entry; 12 months after study entry | 12 months | Finite Gaussian mixture models with LGCM | 3 trajectories for depression:  - high/chronically elevated (38%) - recovery (20%) - low and very low (43%) |
| Wang et al. 2024 China | Prospective cohort | A large-scale cancer centre in Guangzhou | Colorectal | - Mean age: 45.38 ± 8.54 years - Female: 45.3% - Cancer stages: I to IV | Overall distress:  BSI-18 (cut-off ≥ 11) | 214 | 5:  At diagnosis; At discharge; 1 month after surgery; 3 months; 6 months | 6 months | General estimation equation (GEE); GMM and LCGM | From diagnosis to 6 months post-surgery, psychological distress increased at discharge, gradually stabilising thereafter;  4 trajectories for overall distress:  - chronic distress (33.3%) - no distress (41.7%) - steady increase (13.9%) - significantly increased (11.1%) |
| Yang et al. 2024 China | Prospective cohort | 1 provincial cancer hospital and 2 large tertiary general hospitals | Lung | - Mean age: 57.18 ± 11.80 years - Female: 43.87% - Cancer stages: I to III | FCR:  FCRI-SF (cut-off ≥ 13) | 310 | 4: 1 month after surgery (T1); 3 months (T2); 6 months (T3); 12 months (T4) | 1 year | Linear mixed-effects model | FCR changed significantly over time, with a slight decrease during T1–T2, and increase at T3 and gradual decline at T4 |
| Abbreviations: SF-36, Short Form – 36 Health Survey; MCS, Mental Component Score; HADS, Hospital Anxiety and Depression Scale; HADS-A, Hospital Anxiety and Depression Scale – Anxiety Subscale; HADS-D, Hospital Anxiety and Depression Scale – Depression Subscale; DT, Distress Thermometer; FCR, Fear of Cancer Recurrence; CWS, Cancer Worry Scale; CWS-6, Cancer Worry Scale – 6 Items; BSI-18, Brief Symptom Inventory – 18 Items; GSI, Global Severity Index; CES-D, Center for Epidemiologic Studies Depression Scale; STAI, State-Trait Anxiety Inventory; STAI-S, State-Trait Anxiety Inventory – State Anxiety Subscale; STAI-T, State-Trait Anxiety Inventory – Trait Anxiety Subscale; PROMIS, Patient-Reported Outcomes Measurement Information System; GHQ, General Health Questionnaire; ESAS-r; Revised Edmonton Symptom Assessment System; FCRI, Fear of Cancer Recurrence Inventory; VASs, Visual Analogue Scales; PHQ, Patient Health Questionnaire; PHQ-4, Patient Health Questionnaire – 4 Items; FCRI-SF, Fear of Cancer Recurrence Inventory – Short Form; CIPLS, Cancer Problems in Living Scales; FOP-SF, Fear of Disease Progression Short Form. ^†^Mean age was not fully presented in few studies. Summary statistics are based only on studies that provided complete age data. ^‡^The way of determining sample size used for analysis varied across studies. Some studies included only participants with complete data at all assessment time points, while others included all participants who completed baseline assessments for analysis. Summary statistics are reported according to the text. ^§^Information about length of follow-up are not fully presented in few studies that focused on the acute treatment phase. Summary values reflect only studies that provided clear follow-up time information. | | | | | | | | | | |
|  | | | | | | | | | | |
